# Heavier molecular weight ocular viscoelastic devices and timing of post-operative review following cataract surgery

**DOI:** 10.1186/1471-2415-7-2

**Published:** 2007-02-09

**Authors:** Balaji Thirumalai, Trudi L Blamires, Lucenne Brooker, Jon Deeks

**Affiliations:** 1 Department of Ophthalmology, Kettering General Hospital, Rothwell Road, Kettering, NN16 8UZ, UK; 2 Department of Ophthalmology, Northampton General Hospital, Cliftonville, Northampton, NN1 5BD, UK; 3 The Department of Public Health and Epidemiology, The Public Health Building, The University of Birmingham, Birmingham, B15 2TT, UK

## Abstract

**Background:**

To assess the safety of abandoning the next day post-operative review in preference for assessment only 2 hours post-surgery for both phacoemulsification and extracapsular surgery with heavier molecular weight ocular viscoelastic devices (OVD).

**Methods:**

475 patients who underwent uncomplicated cataract surgery using heavier molecular weight ocular viscoelastic device (Healon GV) were studied. Of these 415 were phacoemulsification and 60 extracapsular and none received Intraocular pressure (IOP) lowering prophylaxis at the end of surgery. All were examined at 2 hours post-surgery and on the following day. Results were tabulated and analysed assessing wound stability, corneal clarity, anterior chamber reaction and IOP.

**Results:**

In the time between the two assessments 44 (10.6%) patients developed a total of 53 new problems, with a majority being increases in IOP. Based on the lower threshold of IOP of 30 mmHg, the incidence of new problems at the next-day assessment was 9.8% (95% CI: 7.0 to 13.6) in the phacoemulsification group and 16.3% (7.3 to 29.7)in the extracapsular surgery group. At the higher threshold of IOP of 35 mmHg the corresponding figures were 6.6% and 16.3%.

**Conclusion:**

There is a higher incidence of new problems at the next-day assessment than previous studies with conventional OVD. Therefore results from previous studies using standard OVDs cannot be simply extrapolated to heavier molecular weight OVDs. When these agents are used, routine use of an ocular hypotensive agent may be necessary to increase the safety of abandoning the review on the first post-operative day for phacoemulsification patients. This is to be studied.

## Background

The rising and, in some cases, universal trend towards day case cataract surgery raises a vexed question about the timing of postoperative review. This is particularly true if the aim is to do a "true" day case surgery where the patient is not seen in the hospital the next morning and so goes home on the day of surgery. A number of centres do send patients home on the day of surgery but they are reviewed on the next day either in the hospital or by a home visit by a trained staff member. Previous studies[1-5] have suggested that it is safe for patients to be seen in the immediate few hours after surgery and then to be seen a week later, particularly for uncomplicated phacoemulsification. None of these studies, however, have commented on whether the safety of omitting the review on the first post-operative day surgery persists if a heavier molecular weight ocular viscoelastic device (OVD) is used during the surgery. In this study we assess the safety of abandoning the next day post-operative review in preference for assessment only at 2 hours post-surgery for both phacoemulsification and extracapsular surgery with heavier molecular weight OVD.

## Methods

475 consecutive patients who underwent uncomplicated cataract surgery at our hospital were studied. In all cases, irrespective of the type of cataract extraction, Healon GV was used. Healon GV has a molecular mass of 5 million, with a viscosity at rest of 2.5 million mPas and a elasticity which 10 times higher than the Healon OVD. It is also a very cohesive OVD. The majority 415(87 %) underwent phacoemulsification, while the rest 60(13%) had extracapsular cataract extraction. The phacoemulsification procedure was with a scleral tunnel incision and was a standard procedure using the divide and conquer technique. Meticulous attempts were made to remove as much as possible of the OVD either by automated or manual irrigation-aspiration, using the rock-and-roll technique. Intra-aqueal miochol was routinely used after this. No topical or systemic agent was used prophylactically post-operatively.

None of the patients had any form of Intraocular Pressure (IOP) prophylaxis at the end of surgery. The grade of surgeon varied from Consultant to Senior House Officer, with almost all operated upon by the Consultant.

The patients were examined 2 hours following surgery and on the next day. The 2 hours time interval following surgery was chosen firstly to compare with previous studies[1] and secondly to assist with easing patient discharge on the day of surgery, particularly when they had late afternoon surgery. Parameters assessed included corneal clarity, incision stability and anterior chamber inflammation and these were given a grading on each occasion. IOP was measured in the usual manner using a Goldmann Applanation tonometer. Two cut-off levels of 30 mmHg and 35 mmHg were chosen for analysis of IOP based on previous guidelines/studies[1,5,6] and common practice. Some guidelines and studies[1,6]have indicated that as long as the rise in IOP is only for a short length of time (as is the case after uncomplicated cataract surgery), IOP up to 35 mm Hg does not require active intervention. On the other hand[5], it has been conventional practice to treat IOP of 30 mmHg and above in order to prevent retinal vein occlusions and damage to the optic nerve head[7].

Treatment for adverse events was given as soon as they were first noted. Those that had an IOP above 30 mm Hg were treated to lower the IOP using Acetazolamide or a topical B blocker.

The prevalence of adverse events at the 2 hour and at the next-day assessments was computed and new events occurring between the two assessments identified, and their incidence estimated. Ninety-five percent confidence intervals were computed for incidence rates using the exact binomial method. The significance of differences in rates between phacoemulsification and extracapsular surgery groups was tested using Fisher's exact test.

## Results

Full data on adverse events at both assessments was available on 423 (89%) patients. The prevalence of adverse events at the two assessments is presented in Table 1. Anterior chamber reactions were significantly more common following extracapsular surgery at both assessments (p ≤ 0.001). There were no significant differences (at 2 hours p = 0.45; next day p = 0.35) in problems with corneal clarity or IOP between the surgical groups. Incision instability was rare, but significantly more common after extracapsular surgery at the next-day assessment.

**Table 1:** Prevalence of adverse events

	**Phacoemulsification**		**Extracapsular**		**Total**		**P-value**
	(n = 415)*		(n = 60)†		(n = 475) ‡		(Fisher's exact test)

	n	(%age)	n	(%age)	n	(%age)	

							
**2 hour follow-up**							
Corneal clarity	32	8.1%	6	10.9%	38	8.5%	P = 0.45
Incision stability	2	0.5%	0	0.0%	2	0.5%	P = 1.00
Anterior chamber reaction	25	6.4%	12	22.2%	37	8.3%	P = 0.001
IOP ≥ 30 mmHg	27	7.2%	3	6.0%	30	7.1%	P = 1.00
IOP ≥ 35 mmHg	15	4.0%	0	0.0%	15	3.5%	P = 0.24
							
**Next day follow-up**							
Corneal clarity	21	5.3%	5	9.1%	26	5.8%	P = 0.35
Incision stability	0	0.0%	2	3.6%	2	0.5%	P = 0.02
Anterior chamber reaction	28	7.1%	15	27.8%	43	9.6%	P < 0.001
IOP ≥ 30 mmHg	33	8.8%	4	8.0%	37	8.7%	P = 1.00
IOP ≥ 35 mmHg	15	4.0%	3	6.0%	18	4.3%	P = 0.46
							
**New problems first appearing the next day**							
Corneal clarity	7	1.8%	2	3.6%	9	2.0%	P = 0.31
Incision stability	0	0.0%	2	3.6%	2	0.5%	P = 0.02
Anterior chamber reaction	9	2.3%	4	7.4%	13	2.9%	P = 0.06
IOP ≥ 30 mmHg	26	7.0%	3	6.0%	29	6.9%	P = 1.00
IOP ≥ 35 mmHg	13	3.5%	3	6.0%	16	3.8%	P = 0.42

Numbers evaluable vary between (*) 373 and 393; (†) 50 and 55; (‡) 423 and 448

In the time between the two-hour and next-day assessments 44 (10.6%) patients developed a total of 53 new problems (Table 2). The majority of these problems were increases in IOP (29 showed increases ≥ 30 mmHg and 16 ≥ 35 mmHg) but there were also 13 patients with increased anterior chamber activity, 9 instances of corneal oedema/haze and 2 patients with incision instability. Both cases of incision instability had received phacoemulsification, but otherwise there was no significant difference in the incidence of new problems with type of surgery.

**Table 2:** Incidence of new problems

**Incidence of new problems**	**New cases**	**Incidence rate**	**95% confidence interval**
**Phacoemulsification**			
Any problems (IOP >=30 mmHg)	36	9.8%	(7.0% to 13.6%)
Any problems (IOP >=35 mmHg)	25	6.6%	(4.5% to 9.9%)
			
**Extracapsular**			
Any problems (IOP >=30 mmHg)	8	16.3%	(7.3% to 29.7%)
Any problems (IOP >=35 mmHg)	8	16.3%	(7.3% to 29.7%)
			
**Combined**			
Any problems (IOP >=30 mmHg)	44	10.6%	(7.8% to 14.0%)
Any problems (IOP >=35 mmHg)	33	8.0%	(5.5% to 11.0%)

Based on the lower threshold of IOP of 30 mmHg, the incidence of new problems at the first post-operative day was 9.8% in the phacoemulsification group and 16.3% in the extracapsular surgery group. At the higher threshold of IOP of 35 mmHg the corresponding figures were 6.6% and 16.3%.

## Discussion and conclusion

Heavier molecular weight OVDs have the advantage of maintaining the anterior chamber and affording better protection to the corneal endothelium than standard molecular weight OVDs. The latter property is particularly useful when the patient has a pre-existing corneal dystrophy involving the endothelium or any degree of endothelial decompensation. Though the use of these higher molecular weight OVDs is not widespread for routine cataract surgery, they nevertheless are used by a proportion of surgeons. If any OVD does remain at the end of surgery, this can contribute to increase resistance to aqueous drainage following surgery with a consequent rise of IOP. It is a known that these can remain in the eye to some degree despite meticulous attempts made to aspirate them at the end of surgery. Thus they are the main contributors to resistance to aqueous drainage following surgery, with consequent rise of IOP. As discussed in previous papers retained OVD may cause either trabecular meshwork blockage or post-operative capsular bag hyper distention, anteroplacement of the IOL optic and capsular block from occlusion of the circular opening of the IOL optic[8]. The raised IOP then has a transient bearing on clarity despite increased protection to the corneal endothelium intraoperatively by the OVD.

As evident from the results the proportion of patients requiring intervention on the day following surgery was 10.6%. The rate was slightly lower in the larger phacoemulsification group. The majority of those requiring intervention was due to IOP. Nevertheless in a "true" day case arrangement, the ones that did need intervention would have been missed if they had not been seen on the day following surgery. The Royal College of Ophthalmologists guidelines advises, in view of the very low incidence of endophthalmitis (0.14%), that patients should be pre-warned about the symptoms and that robust arrangements need to be in place to ensure that patients not reviewed the next day should have easy access to advise and assessment should they be symptomatic at any time[9]. While in our study higher molecular weight OVD was used for all patients, other studies have shown results with standard OVDs. A previous study [10] has shown that with conventional OVD usage in uncomplicated phacoemulsification the number of patients requiring intervention/change to their post-operative treatment was 2.2%. Another study[1] has shown that with conventional OVD, 10% of patients required intervention at 2 hours post-operatively for raised IOP (cut-off used ≥ 35 mm Hg) but neither these patients or any new ones required any intervention on the following day. Thus, we have observed a higher incidence of new problems at the next-day assessment than previous studies of conventional OVDs.

At the present time of increasing day case surgery and the huge variety of operative settings, it must be borne in mind that the safety of this type of surgery cannot be taken for granted particularly when greater viscosity OVDs are used. Results from studies using standard OVDs cannot be simply extrapolated. However, given that the majority of identified problems were from raised IOP, there may be a role for using IOP prophylaxis at the end of surgery. The way forward would be to undertake a study wherein patients undergoing uncomplicated cataract surgery with heavier molecular weight OVDs would have to be separated into 2 groups by randomisation. One group receiving IOP lowering prophylaxis at the end of surgery, with the other group as a control and the timing of post-operative review being the same as in this study.

## Competing interests

The author(s) declare that they have no competing interests.

## Authors' contributions

BT Conceived the study, participated in its design, performed surgery, data acquisition, data analysis, literature search, main paper writing and submission

TLB Conceived the study, participated in its design, performed majority of surgery, literature search and paper modifications

LB Data acquisition

JD performed all the statistical analysis

## Pre-publication history

The pre-publication history for this paper can be accessed here:


